# The Association of Serum Hepcidin Levels and Insulin Resistance in PCOS Patients: A Case-Control Study 

**Published:** 2018-12

**Authors:** Batool Hossein Rashidi, Soheila Shams, Mamak Shariat, Maryam Bagheri, Marziyeh Mohebi, Fedyeh Haghollahi

**Affiliations:** 1 Vali-e-Asr Reproductive Health Research Center, Tehran University of Medical Sciences, Tehran, Iran; 2 Maternal, Fetal and Neonatal Research Center, Tehran University of Medical Sciences, Tehran, Iran; 3 Department of Reproductive Health, School of Nursing and Midwifery, Tehran University of Medical Sciences, Tehran, Iran

**Keywords:** PCOS, Hepcidin, Serum Insulin, Insulin Resistance

## Abstract

**Objective:** To investigate the relationship between insulin resistance and hepcidin levels in patients with PCOS.

**Materials and methods:** In this case–control study fifty- six patients with PCOS and forty – one healthy control subjects were included. Plasma levels of hepcidin, IL-6, Serum Insulin and ferritin using ELISA method, serum iron levels using a spectrophotometric method, and Insulin resistance by using HOMA were measured in the two groups of PCOS (case group) and healthy subjects (control group). The results were analyzed by student’s t-test, General Linear Model, Binary logistic and linear regression tests.

**Results:** The mean hepcidin level was 1.97 ± 0.53 pg/ml and 2.40 ± 0.25pg/ml in the case and control groups, respectively. The t-test results showed a significant difference between the two groups (p = 0.0001). The mean of insulin level in case and control group was 30.65 ± 15.02g/dl and 14.71 ± 10.46g/dl, respectively. The t-test analysis indicated a significant difference between the two groups (p = 0.0001). There was an inverse relationship between the level of hepcidin with HOMA-IR (β = -0.629, p = 0.04), and IL-6 (β = -0.243, p = 0.015) in both groups. The adjusted OR proved a statistically significant association between serum hepcidin (OR = 0.063; 95 % CI: 0.01-0.385, p-value  =  0. 003) and HOMA (OR  =  1.569; 95 % CI: 1.254–1.964, p-value  =  0.001) with PolycysticOvarian Syndrome.

**Conclusion:** There was an inverse relationship between hepcidin levels and insulin resistance in both groups meaning decrease in hepcidin levels and increase in insulin resistance may increase the risk of PCOS.

## Introduction

The polycystic ovary syndrome (PCOS) is the most common cause of chronic anovulation which leads to a chronic inflammatory response and hyperandrogenism with a prevalence of 5-10% in the women of reproductive age (1, 2, 3).

The pathophysiology and etiology of this syndrome are complex and still not fully understood (1, 2, 4). It is also hypothesized that insulin resistance is a key factor in the incidence of polycystic ovary syndrome. It has moreover been shown to be four times higher in PCOs than the general population (4).The prevalence of insulin resistance has been reported to range from 20 % to 40% in PCOS (3-5). Insulin resistance and hyperinsulinemia play a pathologic role by increasing ovarian androgen production (6, 7, 8). In a study, the relationship between insulin resistance and iron overload in PCOs were seen which may be related to hepcidin (9).

Hepcidin is an antimicrobial peptide and one of the contributing factors affecting the pathogenesis of PCOSwhich is produced in response to inflammation, elevated serum iron level, and hypoxia (10).Wang et al suggested that hepcidin is directly regulated by insulin and plays an important role in iron overload in diabetic rats (11). A recent study reported that, insulin treatment could be used as a novel method for the correction of hepcidin levels. It moreover can prevent cellular iron overload and reduce the risk of diabetes (12). In another study, hepatic hepcidin mRNA expression in the animal models of insulin-resistant was shown to be lower (13). The finding of other studies revealed that, serum hepcidin levels increase during inflammation and infection which is independent of iron levels. It seems IL-6 plays a critical role in this issue (9, 14).

Serum hepcidin levels in PCOS patients were lower compared to non-PCOs patients (9, 13-15). 

Increased hematopoiesis and decreased expression of the hepcidin gene have been associated with increased iron absorption can lead to a decrease in serum hepcidin levels in response to iron overload in obese PCOSpatients (9, 15).

Hepcidin increases in inflammatory conditions and decreases in cases with hyperandrogenismas well as in cases with insulin resistance (14-18).Therefore, due to the increased inflammatory status, hyperandrogenism, and insulin resistance in PCO (18), this study was conducted to determine the relationship between serum hepcidin levels and insulin resistance in patients with non-obese PCOS.

## Materials and methods

This case-control study was conducted on 97 patients admitted to a university based infertility clinic. After receiving the ethical approval (Reference number 93-01-39-25278, Tehran University of Medical Sciences) and obtaining informed consent from individuals, the project started.

In this study, 56 PCOS women aged from 20 to 40 years, who had referred to the infertility clinic for the first time to treat infertility and who had not received any treatment and blood donation in the preceding year were enrolled as the case group. The control group, on the other hand, consisted of 41 healthy women aged from 20 and 40 years with male factor infertility, normal menstruation, no clinical symptoms or biochemical hyperandrogenism, BMI < 30, normal level of urea, creatinine, and CRP. All demographic data, body mass index and clinical signs of hyperandrogenism, such as acne, hirsutism and male-pattern hair loss, iron and other supplement consumption, as well as other drugs were recorded and evaluated by trained interviewers. 

After obtaining informed consent, all participants undergone a pelvic examination and transvaginal ultrasound in lithotomic position (HS-2600, Honda Electronic Co., LTD, and Japan) with probe frequency 12.5 MHz carried out by a specialist. 10cc brachial vein blood samples were also taken from all subjects by staff clinic. The samples were afterwards sent to alaboratory for testing. Over a period of 4 hours, the blood serum was separated by centrifugation at 3000g for 15 minutes. The samples were stored in a freezer at -70°C until measurement tests. Hepcidin was measured by the ELISA method using the kit (Bioassay Technology-China). 

The sensitivity of the kit was reported to be 5.12pg/ml with the assay range of 10-4000 pg/ml. Normal value of the kit was 0.153-1.888pg/ml. The iron profile was also evaluated for measuring serum iron and ferritin levels. Serum ferritin levels were measured using the kit (Pars Azmoon Co.) and autoanalyzer (BT-3500-Italy). The normal range in the kit was 10-120μg/l.Body mass index (BMI) was calculated by dividing weight by the square of height (kg/cm^2^).BMI was categorized into two groups (≤ 25 and 25-30).

Serum Insulin was measured by the ELISA method using the kit (Monobind-USA). The sensitivity of the kit was 0.72 μU/ml. Normal value in the kit was 0.73 μU/ml. The homeostasis model assessment determines Insulin resistance (IR) (HOMA-IR = [fasting glucose (mg/dl) × fasting insulin (µU/ml)]/405

In order to calculate sample size, the study results from Ramirez et al., 2007 paper was used (14) which evaluated the serum level of hepcidin, ferritin and ferritin/hepcidin in women with PCOS and without PCOS. To compare the mean and standard deviation, 45 samples were required in each group with a power of 80% and α = 0.05.

The present study was carried out from September 2013 to September 2014. The study population consisted of 97 volunteer women living in Tehran (capital city of Iran). The samples were grouped into two groups of PCO and control group (56 women in the PCO group and 41 women in the control group).

After collecting the required information, the data were analyzed using Statistical Package for Social Sciences (SPSS), version 21. Frequency and frequency percentage were calculated for qualitative variables. Means and standard deviations were also determined for quantitative variables. Independent t-test, chi-square, Fisher's exact test were used in the field. The significance level was considered 0.05 in order to interpret the relationships among the variables. Distribution of variables in this study was normal except for ferritin and total testosterone. For normal distributions t-test was utilized. For ferritin and total testosterone the Mann-Whitney test was used. The p-value was also extracted for all distributions. 

The qualitative variables were also compared using chi-square and Fisher exact test. GLM (General Linear Model Univariate) analysis was utilized to evaluate the association between PCOs and categorized BMI on changes of hepcidin level. In order to examine the relation between hepcidin levels and some other effective variables, linear regression analysis was used.

## Results

The mean age in patients was 26.84 ± 4.85 years and 29.39 ± 4.42 in PCOs and control group, respectively. T-test showed that PCOS group was significantly younger than the control group (p ≤ 0.009).

The mean of weight and body mass index were also significantly higher in the case group than the control group (p ≤ 0.006 and p ≤ 0.048, respectively). In total, chi-square test for education, frequency of meat consumption per week, iron tablets and folic acid intake within the last 6 months as well as the t-test analysis for serum iron and ferritin levels showed no significant difference between the two groups, on the contrary, two groups were matched.

The hyperandrogenism symptoms, irregular menstrual cycle and abnormal body mass index (between 25 and 30) in PCOS patients were higher compared to the control group (Table 1).This, however, was expected considering the PCO. The mean of fasting insulin, fasting blood sugar and HOMA-IR Levels were significantly different between two groups (p < 0.05) being higher in PCOs group (Table 1).

The mean hepcidin level was 2.124 ± 0.467pg/ml and 2.327 ± 0.249pg/ml in the case and control groups, respectively. The t-test results indicated a significant difference between the groups (p = 0.013). The iron profile included serum iron, ferritin and ferritin/hepcidin ratio in the two groups. The mean level of IL-6 was 2.39 ± 2.24pg/ml and 2.92 ± 2.67pg/ml in the case and control groups, respectively. The t-test demonstrated no significant difference between two groups (p = 0.311). The mean serum iron level was 72.89 ± 28.97g/dl and 70.62 ± 31.18 g/dl in the case and control groups, respectively (p = 0.539). The mean serum ferritin level was 62.62 ± 42.18 ng/dl and 71.81 ± 100.26 ng/dl in the case and control groups, respectively. As a result of the t-test, PCOS group had higher ferritin/hepcidin ratio (p ≤ 0.019) (Table 1).

Linear regression analysis was used to examine the relationship of hepcidin levels with other effective variables. No significant relationship between the mean of serum ferritin and iron levels with serum hepcidin level of the groups were found. Whereas, HOMA-IR (β = - 0.629; 95 % CI: 0.147-0.002*, *p-value  =  0. 044**)** and Interleukin-6 (β = - 0.243; 95 % CI: 0.088-0.010*, *p-value  =  0 010(were significantly associated with hepcidin level. There was an inverse relationship between the level of hepcidin, HOMA-IR, and IL-6 (Table 2).

GML Univariate analysis showed no interaction between PCOs and BMI that changed on hepcidin level (Data not shown in table, p = 0.089).

Adjusted Multivariate binary logistic regression was utilized to examine the biomarker in relation to odds of PCOs compared to controls.

Potential confounders including maternal age (yrs.), BMI (kg/m^2^), TIBC, Feritin/hepcidin ferritin Interleukin-6,HOMA-IR were included in multivariate models on the basis of the differences between variables in two groups (p-value ≤ 0.05) (Table 2, 3).

The adjusted OR proved a statistically significant association between serum hepcidin (OR = 0.063; 95 % CI: 0.01-0.385*, *p-value = 0. 003) and HOMA (OR  =  1.569; 95 % CI: 1.254–1.964, p-value  =  0.001) as well as the poly cystic ovarian Syndrome (PCOs) (Table 3).

In short, hepcidine levels have an inverse relationship and HOMA has a positive correlation with PCOs meaning that lower levels of hepcidin and higher levels of HOMA- IR increases the risk of PCOs.

**Table 1:** Evaluation of demographic variables (qualitative and quantitative)

	**PCOS** **N (%)**	**Non- PCOS** **N (%)**	**P- value**
o Education:			0.602
1. Primary school	(%32)18	(%34)14	
2. High school	(%48)27	(%44)18	
3. Academic	(%20)11	(%22)9	
o Meat consumption( per week):			0.188
1. No	(%18)10	(%7)3	
2. One to three times	(%77)43	(%81)33	
3. More than three times	(%5)3	(%12)5	
o Taking iron tablets( within last six months:			0.120
1. Yes	(%5)3	(%14.5)6	
2. No	(%95)53	(%85.5)35	
o Taking folic acid( within last six months):			0.964
1. Yes	(%68)38	(%68)28	
2. No	(%32)18	(%32)13	
o Menstrual cycle:			0.0001
1. Regular	(%18)10	(%83)34	
2. Irregular	(%82)46	(%17)7	
o Clinical symptoms of hyperandrogenism:			0.0001
1. Acne	(%21.5)12	(%10.4)4	
2. Hirsutism	(%28.5)16	(%2.5)1	
3. Male pattern hair loss	(%34)19	(%29.5)12	
o Body mass index:			0.0001
1. Normal (< 25)	(%33.9)19	(%53.7)22	
2. Abnormal (25-30)	(%66.1)37	(%46.3)19	
oPCOS ultrasound:			0.0001
1. Yes	(%4.96)54	(%2.4)1	
2. No	(%3.6)2	(%97.6)40	
Age (year)	26.84 ± 4.85	29.39 ± 4.42	0.009
Weight (kg)	69.22 ± 10.18	64.02 ± 6.96	0.006
Body mass index (kg/ m²)	26.52 ± 3.15	25.05 ± 2.51	0.048
Serum iron (μ g/dl)	72.89 ± 28.97	70.62 ± 31.18	0.712
Ferritin (ng/ml)	62.62 + 42.18	71.81 ± 100.26	0.539
Ferritin/hepcidin	34.21 ± 2.77	24.24 ± 16.38	0.019
Hepcidin (pg/ml)	1.97 ± 0.53	2.40 ± 0.25	0.0001
Interleukin -6 (pg/ml)	3.39 ± 2.24	2.92 ± 2.24	0.350
Insuulin (µu/ml)	30.65 ± 15.02	14.71 ± 10.46	0.0001
Fasting blood sugare (mg/dl)	96.37 ± 23.88	89.17 ± 10.65	0.039
HOMA-IR	7.75 ± 4.36	3.29 ± 2.48	0.0001
Total Testestrone (ng/dl)	1.97 ± 9.13	0.68 ± 0.28	0.720

Note: Values are expressed as numbers (%); Mean ± SD

## Discussion

This research was designed and conducted to assess the serum levels of hepcidin and insulin resistance in non-obese PCOS patients and also to evaluate their association. 

The HOMA-IR index was used to evaluate insulin resistance. The present study reported a reverse correlation between serum hepcidin levels and insulin resistance in PCOS patients. The results of a study by Sam AH et al in 2013, showed that insulin resistance had a negative and significant correlation with serum hepcidin levels (13), which was consistent with the findings of the present study. However, a study by Wang H et al in 2013 on rats, revealed that hepcidin decreased by loss of insulin signal and was directly regulated by insulin (11).

The present study is one of the few human studies carried out in this context on non-obese-PCOS patients. Many studies have reported the relation of Insulin resistance and iron levels (11, 14-16, 19-21). Insulin resistance and hyperinsulinemia increase hematopoiesis and down-regulation of hepcidin gene expression, which lead to more iron absorption as well as decrease in serum hepcidin levels (9, 15).

**Table 2:** Linear regression analysis was used to examine relation of hepcidin levels with some variables

**Model**		**Coefficients**	**P.value**	**95,0% Confidence Interval for B**	
		**Beta**		**Lower Bound**	**Upper Bound**
1	(Constant)		0.000	1.876	3.558
	BMI	-0.079	0.436	-0.046	0.020
	ferritin	0.097	0.330	-0.001	0.002
	insulin	0.409	0.188	-0.006	0.032
	HOMA	-0.629	0.044	-0.147	-0.002
	Testestrone2	0.019	0.848	-0.012	0.015
	interlukin6	-0.243	0.015	-0.088	-0.010

Findings of the present study showed no significant relationship between the mean of serum ferritin and iron levels with serum hepcidin level in the two groups (PCO and control groups) (22). In a study conducted by Kim, PCOS patients showed increased serum iron concentration and higher levels of circulating hepcidin compared with those in the control group. According to him, iron excess in lean PCOS women does not lead to defective in hepcidin production (23).

Other studies have shown an inverse relationship between hepcidin and serum iron levels (9, 15, 24, 25). It seems that expression of the hepcidin gene from the iron pathway is responsible for iron absorption and low hepcidin levels in PCOS patients (11, 13, 22, 26).

Hepcidin gene (encoded by Hamp gene) is a 25-amino acid peptide hormone and synthesized in the hepatocyte. It is related to the acute-phase response which is expressed in inflammatory conditions (27, 28, 29).

In this study a reverse association between hepcidin levels with Interlukin-6 in both groups (PCO and control groups) was also found. According to findings of a study by Ganz, infection and inflammation increase the level of interleukin-6 in the body, independent of serum iron levels (28). In the present study, despite the lack of iron levels difference in two groups, serum hepcidin was lower in the PCO group. Perhaps the reason for the reduction of hepcidin in patients with PCOS is the increased interleukin -6 associated with chronic inflammation. Given that similar studies have been carried out on animals, it is highly recommended to conduct more research studies on human beings in order to investigate the relationship between serum hepcidin levels and IL-6 in obese patients with PCOS (28, 30).

There are no known mechanisms for regulating hepcidin in the human brain, moreover the cellular mechanism of hepcidin has not yet been identified (29, 31).

Given the lack of human studies in this field, more comprehensive studies of the hepcidin gene and the pathway of iron absorption using real-time and Western techniques are needed to adequately respond to the causal relationship between hepcidin and insulin resistance. The main limitation of current study appears to be the small and limited number of participants in each group.

**Table 3:** Binary logistic regression analysis was used to examine relation of variable levels in two groups (PCOs and Control)

**Model**		**B**	**Sig.**	**Exp(B)**	**95.0% C.I.for EXP(B)**	
					**Lower**	**Upper**
	BMI	0.042	0.705	1.043	0.839	1.296
	Ferritin	-0.007	0.130	0.993	0.985	1.002
	Hepcidin	-2.759	0.003	0.063	0.010	0.385
	HOMA	0.450	0.000	1.569	1.254	1.964
	Insulin	0.069	0.568	1.072	0.845	1.360
	FBS	-0.004	0.896	0.996	0.941	1.054
	Constant	3.700	0.301	40.431		

As the result, it is suggested to perform further clinical trial studies with larger sample to evaluate insulin resistance and its effect on hepcidin levels in obese PCOS patients. In the current study, the subjects of PCO and control groups were matched for some effective variables. In addition, confounder factors such as the serum interleukin-6, Iron, and ferritin in the two groups were measures in order to determine the effect of these agents on the hepcidin level. Considering that the regulation of hepcidin is multifaceted and complex (25), additional studies are required in understanding the detailed mechanisms of iron sensing that govern hepcidin regulation (25).

## Conclusion

Some association between insulin resistance and hepcidin level in PCOS patients were found. It is recommended to conduct larger, well-designed studies to better evaluate the association between insulin resistance and hepcidin level in PCOS patients for better management of this syndrome.
